# Dental implants: a potential cause of bone marrow edema in the jaw—preliminary report

**DOI:** 10.1186/s40729-021-00306-1

**Published:** 2021-03-29

**Authors:** Hirotaka Muraoka, Naohisa Hirahara, Kotaro Ito, Takumi Kondo, Shungo Ichiki, Takashi Kaneda

**Affiliations:** Department of Radiology, Nihon University School of Dentistry at Matsudo, 2-870-1 Sakaecho-Nishi, Matsudo, Chiba 271-8587 Japan

**Keywords:** MRI, Dental implants, Bone marrow edema, Jaw

## Abstract

**Background:**

Different magnetic resonance imaging (MRI) sequences are frequently used to examine bone marrow in the jaw, including short tau inversion recovery (STIR). MRI is a sensitive method for detecting bone marrow lesions. Currently, pantomography and computed tomography (CT) are used frequently for preoperative dental implant treatment. However, no study has evaluated bone marrow edema around dental implants using MRI. This study aimed to assess bone marrow edema in the jaw around dental implants using brain magnetic resonance images.

**Methods:**

This retrospective cohort study was approved by our university ethics committee (EC19-011). A total of 17 patients (170 sites) who underwent brain MRI between April 2010 and March 2016 were analyzed. All subjects underwent scanning more than 3 years after implant placement. This study investigated two bone marrow signals (with implant site and without implant site). These two groups were then compared using Fisher’s exact test. The Mann–Whitney U test was used to analyze bone marrow signal intensity as the dependent variable and the long and short-axis diameters of the implant as the independent variables.

**Results:**

The were 22/31 sites (71%) and 38/139 sites (27%) of bone marrow edema in the dental implants and without dental implants groups, respectively (*p* < 0.001). Furthermore, there was a significant correlation between bone marrow signal intensity and the short-axis diameter of the implant (*p* < 0.001).

**Conclusion:**

The signal intensity in the bone marrow sites in the jaw with dental implants was significantly higher than that in the sites without dental implants. The present study findings suggest that dental implants are a potential cause of bone marrow edema in the jaw.

## Background

Dental implant prostheses are viable treatment options for both younger and older patients. To perform dental implant treatment, dentists need to know the precise height, width, and contour of the alveolar bone, as well as its relationship with the maxillary sinus and mandibular canal. In clinical settings and for postoperative evaluation, pantomography and computed tomography (CT) are frequently used to evaluate the morphology and quality of the jawbone, and there are many reports about the usefulness of these modalities [1–3].

Bone marrow is a rich cellular connective tissue contained within the bones. Various diseases, such as anemia, inflammatory diseases, leukemia, lymphoma, and metastatic malignant tumors, greatly affect bone marrow and bone function [4, 5]. Moreover, peri-implantitis, affecting the bone marrow around dental implants, causes long-term implant failure, with consequent loss of the implant and the surrounding bone. In recent years, many reports have shown that peri-implantitis, oral squamous cell carcinoma, and bisphosphonate-related osteonecrosis of the jaw (BRONJ) are associated with dental implants [6–9]. Recently, magnetic resonance imaging (MRI) has proven to be a sensitive modality, and owing to its superior spatial resolution, it could assess the bone marrow. MRI is useful for the early detection and assessment of pathologies such as periodontitis and osteomyelitis. However, little attention has been given to the MRI-based evaluation of jawbone marrow signals around dental implants in otherwise healthy patients.

The purpose of this study was to assess bone marrow edema in the jaw around dental implants using MRI.

## Methods

The was a retrospective cohort study and was approved by our university ethics committee (EC19-011). It included 17 patients (12 men, 5 women; 44–77 years of age, mean age, 63.94 years) who underwent brain MRI at the Nihon University School of Dentistry Hospital, Matsudo, Chiba, Japan, from April 2010 to March 2016. Study subjects without implants sites had no clinical findings, such as bleeding on probing (BOP), probing-pocket depth (PD) less than 4 mm, and suppuration (SUPP) around the teeth. Dental implants (φ3.5~5.5×10.0~18.0 mm) and standard implant insertion techniques were used in all subjects, with the implant sites selected 3 years after implant placement. These patients had no pain associated with their implant function, no clinical implant mobility, radiographic alveolar bone loss of less than 2.0 mm, and no history of exudate according to the criteria of Misch et al. [10]. Exclusion criteria included presence of a significant metal artifact, history of radiotherapeutic treatment, and disease (e.g., peri-implantitis, periodontitis, apical periodontitis, tumor or cyst of the jaw) affecting the jawbone marrow.

**Fig. 1:**
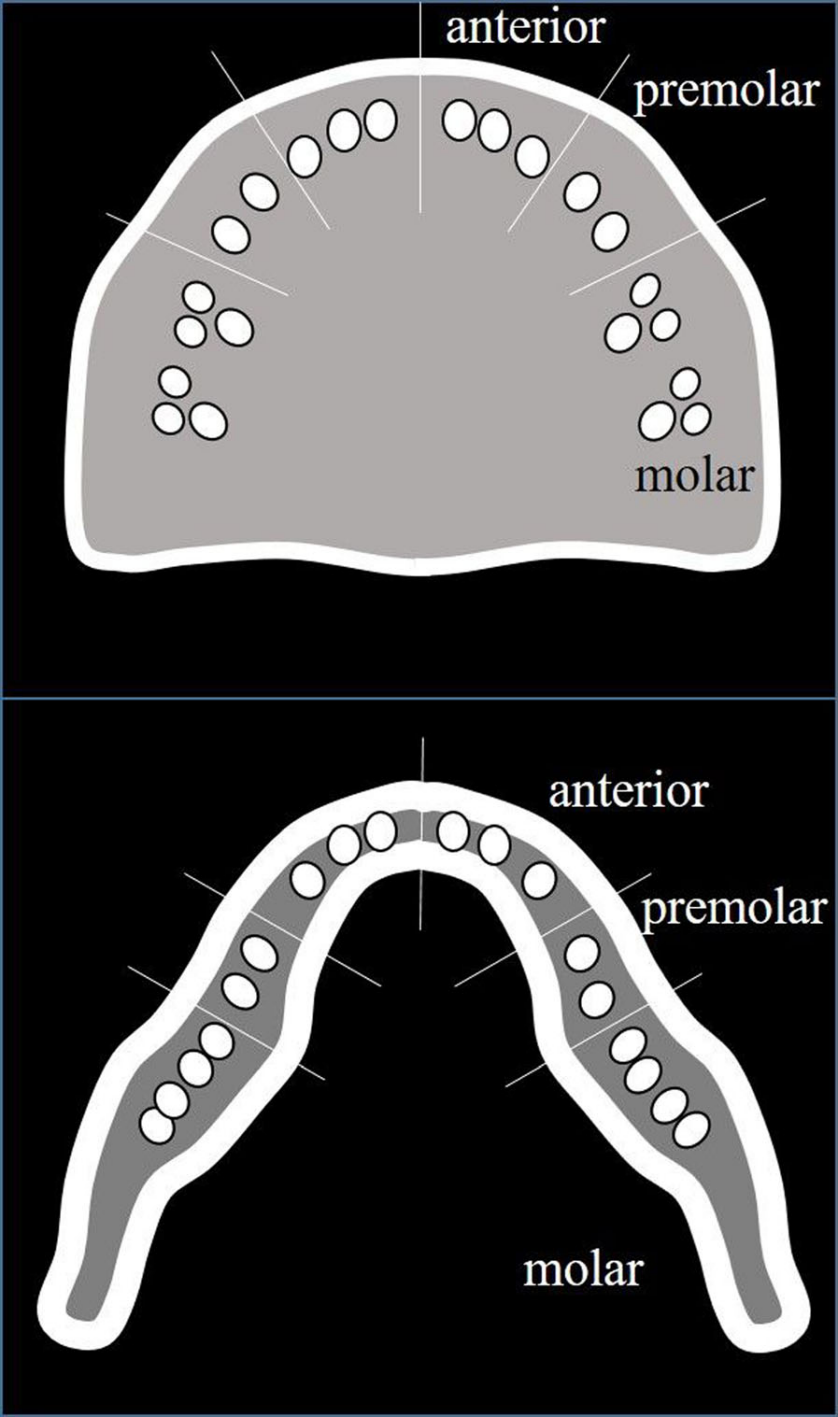
Schema of 12 jaw regions in the axial MR study

**Fig. 2:**
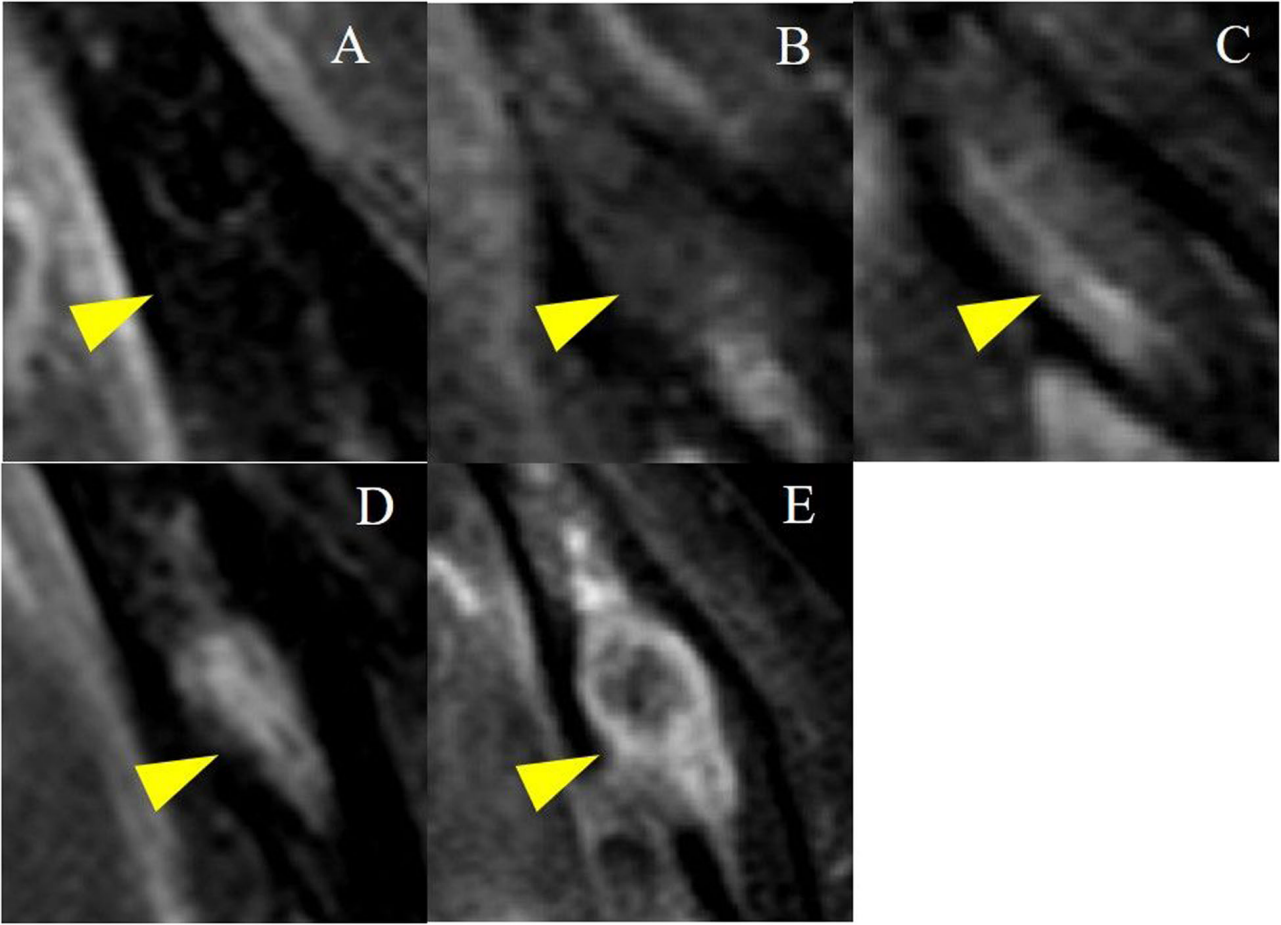
The baseline structures used to determine short tau inversion recovery (STIR) sequence MR signal intensity: **a** fat (low-signal intensity), **b** low-to-intermediate signal intensity, **c** muscle (intermediate signal intensity), **d** intermediate-to-high signal intensity, and **e** cerebrospinal fluid (high-signal intensity) (arrowheads)

MRI was performed using a 1.5-T superconductive MR scanner (Intera Achieva® 1.5 T Nova; Philips Medical Systems, Best, the Netherlands) and a head coil. Short tau inversion recovery (STIR) images were obtained using a spin echo sequence with a repetition time, echo time, and inversion time of 2500, 60, and 180 ms, respectively. Other imaging parameters were set as follow: section thickness, 6 mm; matrix, 320 × 256; field of view, 230 × 195.5 mm; and one acquisition. Images, including those of the jaw, were divided into 12 regions: bilateral anterior, bilateral premolar, bilateral molar in the jaw (Fig. 1). A total of 170 sites (31 sites with implants, 139 sites without implants) were evaluated. These data were independently analyzed by two radiology specialists. The result of Cohen’s kappa statistics was interpreted as follows: values between 0 and 0.2 indicate slight agreement; 0.21–0.39, minimal agreement; 0.40–0.59, weak agreement; 0.60–0.79, moderate agreement; 0.80–0.90, strong agreement; and >0.90, perfect agreement. The baseline components used to evaluate STIR MR signal intensity were cerebrospinal fluid (high signal intensity), muscle (intermediate signal intensity), and fat (low signal intensity). We classified signal intensity into five categories that included intermediate-to-high signal intensity and low-to-intermediate signal intensity in addition to high, intermediate, and low signal intensity (Fig. 2). Normal bone marrow was considered to have low signal intensity. When the bone marrow signal intensity was higher than that of fat, it was considered edematous.

The two groups (with implants site, without implants sites) were then compared using the Fisher's exact test. The Mann–Whitney U test was performed using bone marrow signal intensity as the dependent variable and the long- and short-axis diameters of the implant as the independent variables. These MR signal intensity data analyses were performed using a statistical package (SPSS version 21.0®, IBM Japan Inc., Tokyo, Japan); *p* < 0.05 was considered to indicate significance.

## Results

There was moderate agreement regarding the bone marrow status (Cohen’s kappa = 0.73).

**Fig. 3:**
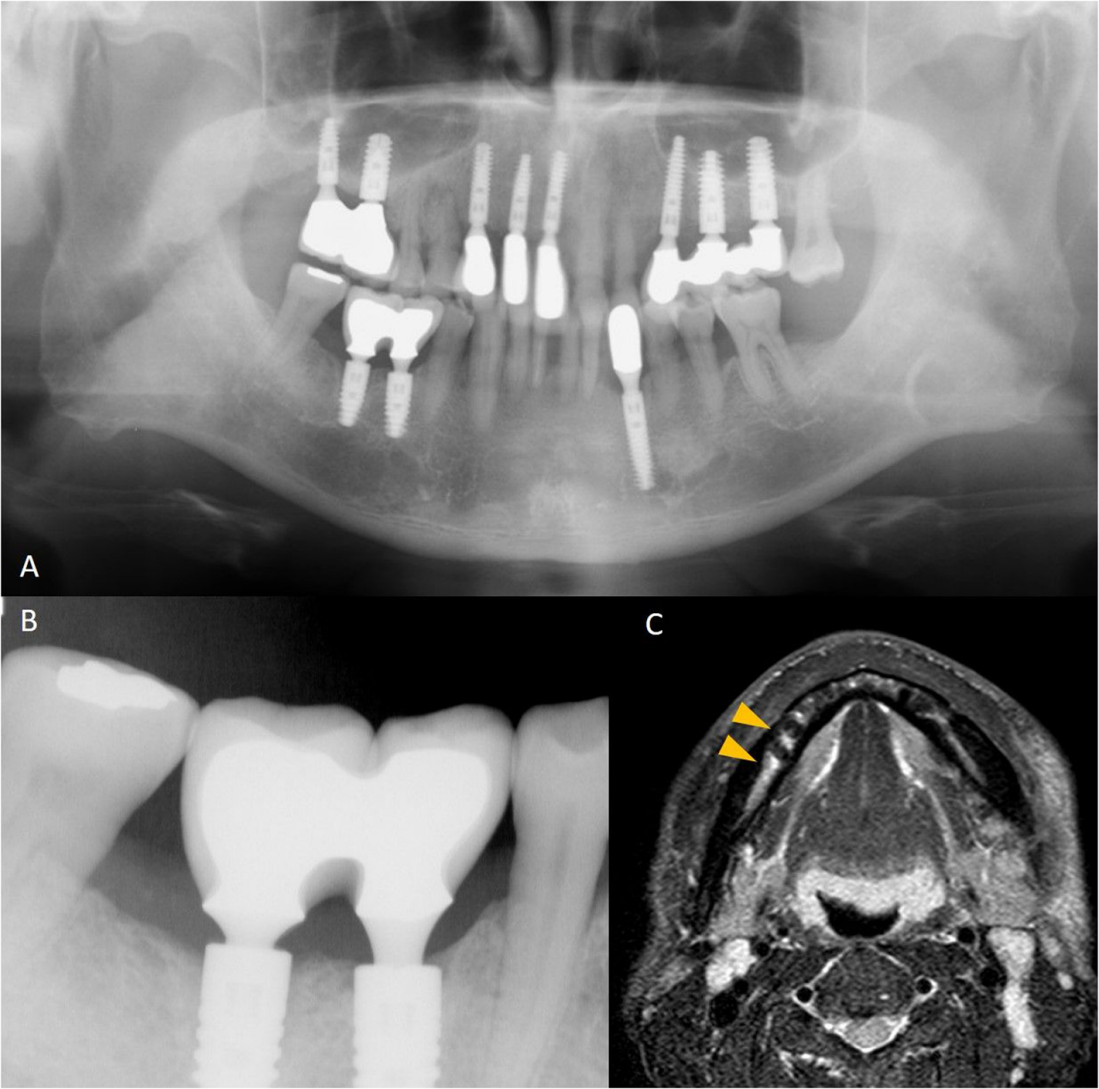
A 61-year-old man with dental implants. This patient had no pain associated with implant function, no clinical implant mobility, less than 2.0 mm of radiographic crestal bone loss, and no history of exudate. **a** Panoramic radiograph shows dental implants embedded in the maxilla and mandible. **b** There are no significant abnormal findings on the intraoral radiograph (arrows). **c** STIR image shows a high-signal intensity on the right side of the mandibular bone marrow around dental implants (arrowheads)

**Fig. 4:**
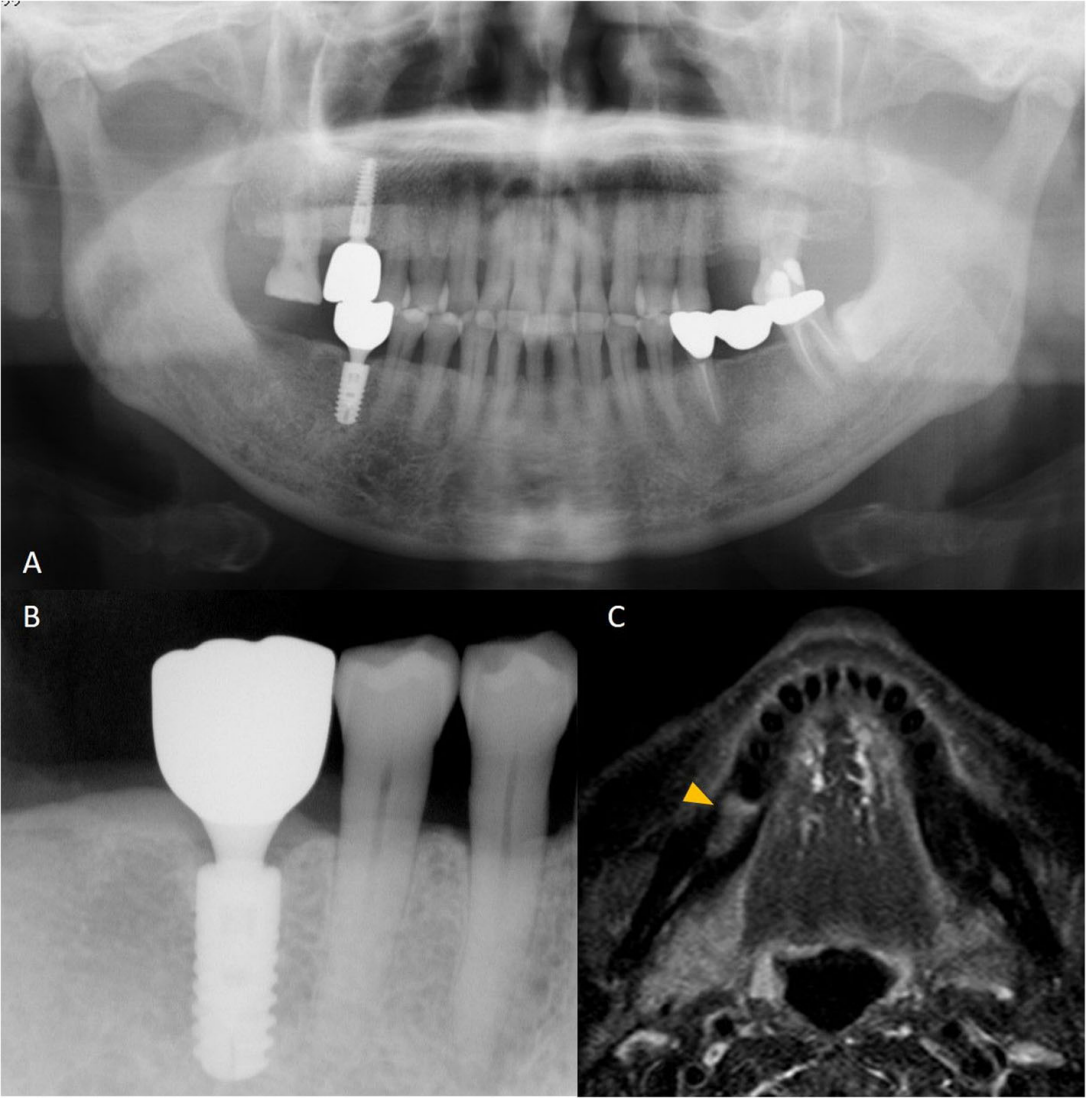
A 73-year-old man with dental implants. This patient had no pain associated with implant function, no clinical implant mobility, less than 2.0 mm of radiographic crestal bone loss, and no history of exudate. **a** Panoramic radiograph shows dental implants embedded in the maxilla and mandible. **b** There are no significant abnormal findings on the intraoral radiograph (arrows). **c** STIR image shows a high-signal intensity on the right side of the mandibular bone marrow around the dental implant (arrowheads)

Table 1 shows the details of each implant size and bone signal intensity. Table 2 shows the bone marrow status in the presence and absence of dental implants. The bone marrow signal intensity was significantly higher in the with dental implants group than in the without dental implants groups. There were no significant abnormal findings on panoramic or intraoral radiographs (Figs. 3 and 4). There were 22/31 sites (71%) and 38/139 sites (27%) with bone marrow edema in the with dental implants and without dental implants groups, respectively (*p* < 0.001).

**Table 1:** Details of each implant size and bone signal intensity

Implant No.	Bone signal intensity	Long axis diameter (mm)	Short axis diameter (mm)
**1**	Low	17	3.5
**2**	Low	14	3.5
**3**	Low	17	3.5
**4**	Low	14	4.5
**5**	Low	14	4.1
**6**	Low	13	4
**7**	Low	15	4
**8**	Low	16	4.2
**9**	Low	15	4
**10**	Low-to-intermediate	15	5
**11**	Low-to-intermediate	18	4
**12**	Intermediate	14	5
**13**	Intermediate	14	5.5
**14**	Intermediate	14	5.5
**15**	Intermediate	14	5.5
**16**	Intermediate	14	4.5
**17**	Intermediate	14	5
**18**	Intermediate	15	5
**19**	Intermediate	15	5
**20**	Intermediate	14	5
**21**	Intermediate	14	5
**22**	Intermediate	13	5
**23**	Intermediate	13	5
**24**	Intermediate	14	5.5
**25**	Intermediate	17	4.5
**26**	Intermediate	12	4.8
**27**	Intermediate	10	5
**28**	Intermediate	14	4.5
**29**	Intermediate	14	5.5
**30**	Intermediate-to-high	14	5
**31**	Intermediate-to-high	12	4.7
**32**	Intermediate-to-high	14	5.5

**Table 2:** Bone marrow edema in the jaw

	Bone marrow edema			*P* values
	presence	absence	Total	
With dental implant	22 (71%)	9 (29%)	31 (18%)	*p* < 0.001
Without dental implant	38 (27%)	101 (73%)	139 (82%)	
Total	60 (35%)	110 (65%)	170

There were 9/31 sites (29%) and 101/139 sites (73%) without bone marrow edema in the with dental implants and without dental implants groups, respectively (*p* < 0.01). All patients in the with dental implant group had no pain associated with implant function, no clinical implant mobility, less than 2.0 mm of radiographic crestal bone loss, and no history of exudate.

Table 3 shows the correlation between bone marrow signal intensity and the long- and short-axis diameters of the implant. There was a significant correlation between bone marrow signal intensity and the short-axis diameter of the implant (*p* < 0.001).

**Table 3:** Relationship between the bone marrow edema and implant size in the around dental implants

		Bone marrow edema		*P* values
		Presence (signal intensity = low-to-intermediate to high)	Absence (signal intensity=low)	
**Implant size**				
**Mean short axis diameter ± SD (mm)**		5.0 ± 0.39	3.92 ± 0.35	< 0.001
**Mean long axis diameter ± SD** **(mm)**		14.0 ± 1.57	15.0 ± 1.41	.124

*SD =* Standard deviation

## Discussion

In this study, significant differences were observed in the jawbone marrow signal intensity between the with dental implants and without dental implants groups.

The jaw contains a rich supply of bone marrow, which is a semi-solid tissue that may be found within the spongy or cancellous portions of bones. At birth, the mandible only contains red bone marrow; therefore, there is an overall low signal intensity on MRI. As a child grows older, conversion of the red marrow to yellow marrow begins anteriorly and proceeds toward the molar regions, angle, and condyle, in that order [11]. Various diseases can greatly affect bone marrow and its function. Therefore, evaluation of bone marrow is critical for the diagnosis, treatment, and prognosis of various diseases [5]. Frequently, problems arise in determining whether an observed MRI bone marrow pattern is normal or abnormal. MRI is an important routine diagnostic tool used for imaging the oral maxillofacial area. STIR imaging represents a useful tool for the evaluation of marrow disease [12]. This study reported a characteristic high sensitivity of MRI for detecting edema. Past studies of MRI-based pre-surgical dental implant assessment have reported on the influence of dental materials on dental MRI [13–15]. However, no study has investigated the jawbone marrow around the dental implant using MRI.

**Fig. 5:**
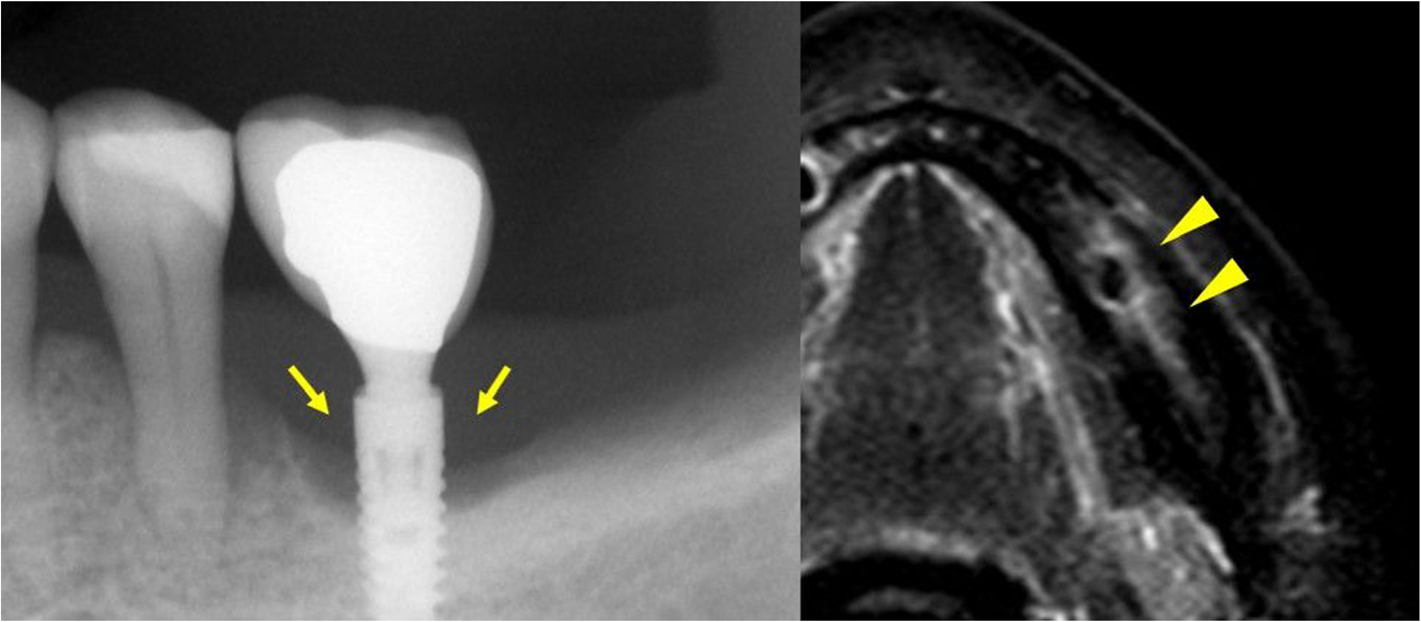
A 66-year-old woman with peri-implantitis. This patient had status such as more than 2.0 mm of radiographically crestal bone loss (arrows), significant bone marrow edema (arrowheads)

Peri-implantitis is defined as an inflammatory process affecting the tissues around an osseointegrated, functional implant, resulting in the loss of the supporting bone [16]. This disease is diagnosed by changes in probing depth and radiographic evidence of bone destruction, suppuration, calculus buildup, swelling, color change, and bleeding. To diagnose an infected implant site, soft tissue measurements using probes have been suggested. Periodontitis and peri-implantitis show characteristic bone marrow edema on MRI, and absence of bone marrow edema indicates that there are no abnormal clinical findings [17]. Muramatsu et al. reported bone marrow abnormalities in a high percentage of MR images of the mandibles of patients with periodontitis [18]. Generally, remarkable bone marrow edema is seen in peri-implantitis with clinical findings, such as bleeding on probing and more than 2.0 mm of radiographic crestal bone loss (Fig. 5). In this study, characteristic bone marrow edema was seen despite the lack of clinically abnormal findings. It was considered that these changes in bone marrow edema occurred because of factors, such as the initial phase of peri-implantitis and occlusal trauma despite no abnormalities observed clinically at the implant placement site. This finding also suggests that bone marrow edema may be more likely to occur in implants without a periodontal ligament with stem cells.

Second, another factor that was considered, which demonstrates spontaneous occurrence, was squamous cell carcinoma. Previous reports have indicated that squamous cell carcinoma around dental implants has been reported to cause chronic inflammation [8, 9]. Another discovery of this study was that there was a significant correlation between bone marrow signal intensity and the short-axis diameter of the implant. This finding may reflect that the larger the short-axis diameter, the more susceptible it is to bacterial colonization. Therefore, long-term evaluation of bone marrow is important. The present study suggested the usefulness of MRI-based examination of dental implants, and may contribute to the development of postoperative follow-up protocols.

However, our study has a few limitations. First, the sample size in our study was small due to the retrospective observational study design. We believe that long-term follow-up studies are necessary to obtain conclusive results. Moreover, STIR images are image sequences that are less affected by magnetic susceptibility artifacts; however, it was not possible to measure the bone signal intensity in patients with severe image distortions resulting from susceptibility artifacts induced by the implant material.

In conclusion, the signal intensity in the bone marrow sites with dental implants was significantly higher than that in the sites without dental implants. The present study suggests that bone marrow edema is caused by dental implants, and that MRI may be useful for evaluating the initial phase of peri-implantitis.

## Data Availability

Not applicable.

## Abbreviations

MRI: Magnetic resonance imaging
STIR: Short tau inversion recovery
CT: Computed tomography
BRONJ: Bisphosphonate-related jaw osteonecrosis
BOP: Bleeding on probing
PD: Probing-pocket depth
SUPP: Suppuration
SD: Standard deviation
